# One *NF1* Mutation may Conceal Another

**DOI:** 10.3390/genes10090633

**Published:** 2019-08-22

**Authors:** Laurence Pacot, Cyril Burin des Roziers, Ingrid Laurendeau, Audrey Briand-Suleau, Audrey Coustier, Théodora Mayard, Camille Tlemsani, Laurence Faivre, Quentin Thomas, Diana Rodriguez, Sophie Blesson, Hélène Dollfus, Yvon-Gauthier Muller, Béatrice Parfait, Michel Vidaud, Brigitte Gilbert-Dussardier, Catherine Yardin, Benjamin Dauriat, Christian Derancourt, Dominique Vidaud, Eric Pasmant

**Affiliations:** 1 Service de Génétique et Biologie Moléculaires, Hôpital Cochin, HUPC, Assistance Publique-Hôpitaux de Paris, 75014 Paris, France; 2 Institut Cochin, INSERM U1016, Université Paris Descartes, 75014 Paris, France; 3 Inserm, UMR 1231, Génétique des Anomalies du Développement, Université de Bourgogne, 21079 Dijon, France; 4 Centre de Référence Anomalies du Développement et Syndromes Malformatifs, Hôpital d’Enfants, 21079 Dijon, France; 5 Department of Child Neurology and National Reference Center for Neurogenetic Disorders, Armand Trousseau Hospital, GHUEP, AP-HP, INSERM U1141, 75012 Paris, France; 6 GRC n°19 ConCer-LD, Sorbonne Université, 75012 Paris, France; 7 Service de Génétique, CHRU de Tours, 37044 Tours, France; 8 Centre de référence pour les Affections Rares en Génétique Ophtalmologique (CARGO), Hôpital Civil, 67091 Strasbourg, France; 9 Service de Génétique Médicale, Hôpital de Hautepierre, 67200 Strasbourg, France; 10 Laboratoire de Génétique Médicale, INSERM U1112, 67000 Strasbourg, France; 11 Service de Génétique, EA3808, Université de Poitiers, CHU de Poitiers, 86000 Poitiers, France; 12 Department of Cytogenetics and clinical genetics, Limoges University Hospital, 87042 Limoges, France; 13 UMR 7252, Limoges University, CNRS, XLIM, 87000 Limoges, France; 14 EA 4537, Antilles University, 97261 Fort-de-France, Martinique, France; 15 DRCI, Martinique University Hospital, 97261 Fort-de-France, Martinique, France

**Keywords:** de novo variant, Legius syndrome, neurofibromatosis type 1, *NF1*, *SPRED1*

## Abstract

Neurofibromatosis type 1 (NF1) is an autosomal dominant disease with complete penetrance but high variable expressivity. NF1 is caused by loss-of-function mutations in the *NF1* gene, a negative regulator of the RAS-MAPK pathway. The *NF1* gene has one of the highest mutation rates in human disorders, which may explain the outbreak of independent de novo variants in the same family. Here, we report the co-occurrence of pathogenic variants in the *NF1* and *SPRED1* genes in six families with NF1 and Legius syndrome, using next-generation sequencing. In five of these families, we observed the co-occurrence of two independent *NF1* variants. All *NF1* variants were classified as pathogenic, according to the American College of Medical Genetics and Genomics and the Association for Molecular Pathology (ACMG-AMP) guidelines. In the sixth family, one sibling inherited a complete deletion of the *NF1* gene from her mother and carried a variant of unknown significance in the *SPRED1* gene. This variant was also present in her brother, who was diagnosed with Legius syndrome, a differential diagnosis of NF1. This work illustrates the complexity of molecular diagnosis in a not-so-rare genetic disease.

## 1. Introduction

Neurofibromatosis type 1 (NF1; MIM#162200) is an autosomal dominant disease with a worldwide incidence between 1 in 2500 and 1 in 3000 individuals [1]. Clinical features predominantly consist of multiple café-au-lait spots (CALS), axillary or inguinal freckling, Lisch nodules, and neurofibromas (NFs). Other symptoms can develop in some patients, such as optic pathway gliomas (OPGs), short stature, or pseudarthrosis. Affected patients are predisposed to benign peripheral nervous tumors [2] called plexiform neurofibromas (PNFs), which can transform into malignant peripheral nerve sheath tumors (MPNSTs) in ~10% of cases [3]. The NF1 diagnosis relies on the National Institutes of Health (NIH) clinical criteria [4]. NF1 is characterized by complete penetrance and great inter- and intrafamilial variable expressivity [5].

More than 95% of NF1 cases [6] are caused by loss-of-function mutations in the *NF1* gene (MIM*613113), which is located at 17q11.2. *NF1* comprises 57 constitutive and three alternative exons and spans over ~350 kb. The 8454-nucleotide open reading frame of the *NF1* gene (NM_00267.3) encodes a 2818-amino-acid protein, neurofibromin, which shows tumor suppressor function [7] by negatively regulating the RAS-MAPK pathway [8]. The *NF1* gene shows one of the highest mutation rates [9], with more than 2600 different pathogenic variants referenced in public databases (Leiden Open Variation Database and Human Gene Mutation Database). About 50% of all NF1 cases are sporadic, i.e., due to de novo mutations [10,11,12].

Some of the NF1 clinical features are common to other diseases, such as Legius syndrome (MIM#611431), an autosomal dominant disorder characterized by the presence of multiple CALS, sometimes associated with skinfold freckling and macrocephaly or learning disabilities. Legius syndrome is caused by loss-of-function mutations in the *SPRED1* gene (MIM*609291) [13,14,15]. The SPRED1 protein acts as a negative regulator of the RAS-MAPK pathway [16].

Here, we report the observation of six NF1 families. Surprisingly, the *NF1* pathogenic variant identified in the index cases was not present in all the other affected members of the family, thus revealing the presence of a second independent *NF1* pathogenic variant in the same family. In one family, NF1 and Legius syndrome co-occurred in siblings, challenging the molecular diagnosis for these patients. Our study aimed at highlighting the complexity of molecular diagnosis in NF1 families where patients can carry distinct pathogenic variants or may present with phenocopies.

## 2. Materials and Methods

### 2.1. Study Samples

Informed consent was collected for all the patients and their relatives (ethical approval code: *Comités de protection des personnes* CPP17/79, A0296746, and 2015-08-11DC). Samples were collected on EDTA tubes for DNA analysis and on PAXgene™ Blood RNA Tubes (Becton Dickinson, Rungis, France) for RNA analysis. All patients underwent a physical examination with a referent clinician who recorded the phenotypic data according to a standardized questionnaire including: number and size of CALS; number and localization of freckling; number and localization of plexiform and spinal neurofibromas; number of cutaneous and subcutaneous neurofibromas, each classified into quantitative categories of 0, 1–9, 10–99, and >100; the presence or absence of MPNSTs, Lisch nodules, optic pathway gliomas, skeletal malformations, or developmental delay.

### 2.2. DNA and RNA Extractions

DNA extraction was performed with the Maxwell^®^ 16 LEV Blood DNA Kit (Promega, Charbonnières-les-Bains, France) on EDTA blood samples according to the manufacturer’s instructions. RNA was directly extracted from 5 mL of PAXgene^TM^ blood samples with the PAXgene^TM^ Blood RNA System (Qiagen, Courtaboeuf, France) according to the manufacturer’s instructions. DNA concentrations were quantified using a NanoDrop 1000 Spectrophotometer v3.8 (Thermo Fisher Scientific, Courtaboeuf, France).

### 2.3. Microsatellite Typing

Four *NF1* intragenic polymorphic microsatellites (D17S1307, D17S2163, D17S1166, and GDB:270136) were studied for segregation analysis. Three additional *NF1* extragenic polymorphic microsatellites (D17S841, D17S1800, and D17S798) were also genotyped when the previous ones were not informative. Three *SPRED1* intragenic polymorphic microsatellites were studied for family 6 (19AT, 19TG, 23AC). DNA samples were diluted to a concentration of 10 ng/mL and amplified using dedicated primers and the Taq GOLD polymerase (Thermo Fisher Scientific). The GS-500LIZ (Thermo Fisher Scientific) marker was used for detection. Sequencing analysis was performed on an ABI Prism 3130 automatic DNA sequencer (Thermo Fisher Scientific). The results were analyzed with the GeneMapper^®^ v.4.0 software package (Thermo Fisher Scientific). Samples with a unique haplotype were tested for large *NF1* deletion using real-time PCR-based gene dosage of exons 8 and 57, as described in Sabbagh et al., 2013 [6]. To confirm that variants were de novo, both maternity and paternity were confirmed using a PowerPlex^®^ 16 HS System (Promega, Madison, WI, USA) kit to analyze the segregation of 15 short tandem repeat (STR) markers.

### 2.4. NF1 and SPRED1 Next-Generation Sequencing (NGS)

Experiments were performed at the NGS facility of Cochin Hospital, Paris (Assistance Publique-Hôpitaux de Paris, France). The techniques were previously described by Pasmant et al., 2015 [17]. Variants were analyzed with NextGENe v.2.4.2.2 (Softgenetics, State College, PA, USA) and Polydiag (Imagine Institute, Necker Hospital, Paris, France) software. 

### 2.5. DNA and RNA Sequencing with PCR

Polymerase chain reaction (PCR) was performed either for screening purposes or for confirmation of NGS results on cDNA after reverse transcription of the *NF1* transcript, or on DNA samples, respectively. Selected exons of the *NF1* (NM_00267.3) and *SPRED1* (NM_152594.2) genes were amplified by PCR and sequenced as previously described in Sabbagh et al., 2013 [6]. Variants identified by cDNA or NGS approaches were confirmed using DNA sequencing analysis performed on the corresponding exon. Sequences were aligned on the reference sequence with Seqscape analysis software v2.5 (Thermo Fisher Scientific). 

### 2.6. Variant Classification

Variants in the *NF1* and *SPRED1* genes are reported according to the reference sequences NM_00267.3 and NM_152594.2, respectively, with the first nucleotide of the first methionine codon corresponding to the +1 position. Exon numbering is indicated according to the NCBI nomenclature. Variants were named at the coding DNA and protein levels according to the Human Genome Variation Society (HGVS) recommendations. In silico predictions of the effect of missense variants were performed with SIFT, MutationTaster, and Polyphen-2, together with SpliceSiteFinder-like, MaxEntScan, NNSPLICE, GeneSplicer, and Human Splice Finder embedded in Alamut visual software v.2.7.1 (Interactive Biosoftware, Rouen, France). CADD scores were obtained for single nucleotide variants (SNVs) with the 1.4 version. Variant frequency in the general population was checked in the gnomAD and dbSNP databases. The Leiden Open Variation Database (LOVD) and ClinVar databases were systematically checked for all variants. An assessment of variants’ pathogenicity was performed according to the American College of Medical Genetics and Genomics and the Association for Molecular Pathology (ACMG-AMP) guidelines [18]. 

## 3. Results

**Figure 1:**
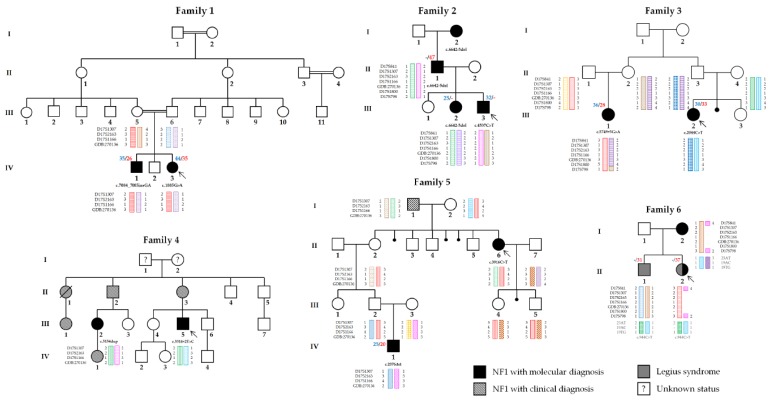
Pedigrees of the six NF1 families. NF1 patients with molecular diagnosis, relatives reported as presenting a NF1 phenotype, and patients with a Legius syndrome are shown in black, black crosshatching, and gray, respectively. Haplotypes for the *NF1* and *SPRED1* loci are depicted for the corresponding patients. Arrows indicate the propositus in each family. Paternal and maternal ages at conception are indicated above the patient in blue and red, respectively, when available.

Pedigrees and clinical data of NF1 patients are shown in Figure 1 and Table 1, respectively. Ages at last examination are indicated in the corresponding column. All variants were classified according to the ACMG-AMP guidelines [18] (Table 2).

Family 1 is a Sephardic family in which two siblings (patients IV-1 and IV-3) were clinically diagnosed with NF1. The first child of the kindred (IV-1) had more than eight CALS >1.5 cm and five CALS <1.5 cm. He also had one subcutaneous NF on the right forearm, an anemic nevus on the left thigh, bilateral Lisch nodules, mild scoliosis, and learning disabilities with a global IQ of 105 at the age of 16. His sister (IV-3) had a Sylvian artery stroke when she was eight months old, leading to permanent left hemiparesis. She was diagnosed with NF1 as she had more than six CALS > 1.5 cm and multiple smaller CALS, bilateral axillary and inguinal freckling, cutaneous NFs, bilateral Lisch nodules, and mild scoliosis. The third child of the kindred (IV-2) suffered from intellectual disabilities, including reading delays. However, he did not show any sign of NF1. The family history revealed the existence of cysts in several relatives (eyelid cysts in III-7, head cysts in III-8, and renal cysts in III-10), but no formal diagnosis of NF1 was made in any of them. Molecular diagnosis in the two siblings IV-1 and IV-3 revealed two independent pathogenic variants in the *NF1* gene: c.7084_7085insGA (p.Asn2362Argfs*14) and c.1885G>A (p.Gln616Glyfs*4), respectively. Sequencing of the parents’ DNA confirmed that both pathogenic variants occurred de novo. To determine the parental origin of each variant, long-range PCR was performed in the parents and children. Sequencing of the distal part of the intron 48 revealed the presence of the c.7126+37C>G variant in both the father (II-6) and his affected son (IV-1). This benign variant was in *cis* with the c.7084_7085insGA pathogenic variant found in the affected son (IV-1), indicating that the de novo c.7084_7085insGA pathogenic variant occurred in the *NF1* paternal allele.

Family 2 is a Caucasian family with a medical history of NF1 in the paternal branch. All the affected members of family 2 (patients I-2, II-1, III-2, and III-3) had more than six CALS. The grandmother, I-2, was also described as having developed a scalp tumor. The propositus (III-3) had a more severe clinical presentation including bilateral axillary freckling and Lisch nodules, OPG, multiple cutaneous and subcutaneous NFs, and a thoracic PNF. He also developed a spinal NF near the left iliac crest, xanthogranulomas, scoliosis, and suffered from attention deficit disorder. NGS revealed the presence of the c.4537C>T, p.(Arg1513*) pathogenic variant in the *NF1* gene, which was confirmed at the transcript level. This variant was not found in the other affected members of the family. The grandmother (I-2), father (II-1), and sister (III-2) of the propositus carried the c.6642-5del pathogenic *NF1* variant, which is responsible for the exon 45 skip in the *NF1* transcript: p.Phe2215Hisfs*6.

In family 3, the propositus (patient III-2) presented with an OPG associated with CALS. NGS analysis in the propositus identified the c.2044C>T, p.(Gln682*) pathogenic variant in the *NF1* gene. Her cousin (III-1) showed multiple CALS, bilateral sub-mammal freckling, cutaneous and subcutaneous NFs, one PNF, and scoliosis. Unidentified bright objects (UBOs) were revealed on MRI, but no OPG was identified. NGS and *NF1* transcript analysis identified the c.5749+5G>A, p.Ser1850fs*2 pathogenic variant in the *NF1* gene (leading to the exon 39 skip in the *NF1* transcript). Parental DNA studies showed the de novo origin of both variants in patients III-1 and III-2. Segregation analysis revealed that the two patients inherited distinct NF1 haplotypes from their respective parents, suggesting that the two variants occurred on different alleles of the *NF1* gene.

Family 4 is a Caucasian family with a medical history of NF1. The propositus (patient III-5) was a 39-year-old man presenting with CALS, bilateral axillary freckling, Lisch nodules, and cutaneous and subcutaneous neurofibromas. In addition to NF1, he also suffered from Brugada syndrome (MIM#601144), inherited from his father. NGS analysis of *NF1* in patient III-5 revealed the c.3314+2T>C *NF1* pathogenic variant, which was predicted by in silico tools to alter the *NF1* transcript splicing. His cousin (patient III-2), a 26-year-old woman, was addressed for a molecular confirmation of her clinical NF1, including CALS, axillary freckling, and cutaneous NFs. NGS analysis revealed the c.5154dup, p.(Phe1719Ilefs*17) *NF1* pathogenic variant in patient III-2. NF1 symptoms were mentioned in several members of the family, including their respective parents (patients II-3 and II-2), an aunt who died from a brain tumor (II-1), and her daughter (III-1).

The propositus of family 5 (patient II-6) was a 23-year-old girl with short stature (150 cm), multiple CALS, axillary freckling, cutaneous and subcutaneous NFs, one plexiform neurofibroma in the lumbar fossa, Lisch nodules, and scoliosis. She had developed breast cancer. NGS analysis identified the c.3916C>T, p.(Arg1306*) *NF1* pathogenic variant. Her great-nephew (patient IV-1) was also diagnosed with NF1. He presented a unilateral OPG at the age of two. He suffered from pseudarthrosis and sphenoid wing dysplasia. He underwent multiple surgeries for a tibial deformation. Together with the classical manifestations of the disease, both patients II-6 and IV-1 showed UBOs on MRI. Patient IV-1 carried a different *NF1* pathogenic variant from the propositus: c.2376del, p.Asn793Thrfs*28. Molecular analysis of the family showed that both *NF1* variants occurred de novo. Of note, the father of the propositus (individual I-1) was described as affected by NF1 (presenting Lisch nodules and CALS), but he did not carry his daughter’s *NF1* variant, nor any other *NF1* variant that could have been detected by our techniques.

In family 6, both the mother (patient I-2) and the daughter (II-2) carried a large deletion of the *NF1* locus. The 10-year-old propositus (II-2) presented multiple CALS, freckling, cutaneous NFs, and a cardiac malformation. Her mother (I-2) presented CALS, cutaneous NFs, and scoliosis. The propositus’ brother (II-1) only showed multiple CALS and freckling. No pathogenic variant or deletion of the *NF1* gene could be detected in this patient. Real-time quantitative PCR was performed in all three patients and confirmed the absence of deletion at the *NF1* locus for II-1. However, he and his sister both carried a variant of unknown significance in the *SPRED1* gene: c.944C>T, p.(Pro315Leu). The variant was not detected in their mother, but their father could not be tested. The Pro315 is evolutionarily conserved and proline presents very different chemical and physical properties from leucine. The c.944C>T, p.(Pro315Leu) variant is rare in the global population (31/282544 alleles in gnomAD) and is predominantly found in the African population (30/24958).

## 4. Discussion

Because of its great phenotypic and genetic heterogeneities, NF1 diagnosis can be challenging. To date, few cases of two or more different *NF1* pathogenic variants in the same family have been described [6,23,24]. Such cases are challenging and the absence of a familial mutation in a patient presenting with NF1 clinical features is not sufficient to exclude the diagnosis. On the contrary, the high mutation rate reported in the *NF1* gene [9] should encourage us to search for the occurrence of a de novo mutation in any suspected case.

Likewise, phenocopies in a family can lead to a delayed diagnosis. As mentioned above for family 6, Legius syndrome can be mistaken for NF1. The inability to find a *NF1* pathogenic variant should encourage the exploration of the *SPRED1* gene when confronted with a mild phenotype. In the case of family 6, the pathogenicity of the *SPRED1* c.944C>T variant could not be ascertained. Its allelic frequency (31/282544 alleles in GnomAD, predominantly in the African population) and the lack of data about its de novo origin questioned its role in the development of CALS in patient II-1. The *SPRED1* c.944C>T, p.(Pro315Leu) variant was thus classified as a variant of unknown significance according to the ACMG-AMP guidelines. Only one case of co-occurrence of NF1 and Legius syndrome in the same family (with confirmed pathogenic variants in the *NF1* and *SPRED1* genes) has previously been reported [25]. Moreover, some *NF1* mutations can lead to the development of the overlapping neurofibromatosis‒Noonan syndrome (MIM #601321) [26,27,28,29,30,31], which combines the typical features of NF1 (CALS, axillary and inguinal freckling, Lisch nodules, OPG, and neurofibromas) and Noonan syndrome (short stature, facial dysmorphism, and congenital heart defects, among others). This observation further reduces the gap between RASopathies and complicates genetic counseling, even more so when NF1 and Noonan syndrome co-exist in one family [32,33] or in one patient [34,35,36].

Studies have suggested that most de novo point mutations or small indels have a paternal origin [37], whereas large deletions are predominantly of maternal origin, as is the case with NF1 [38]. Advanced paternal age can substantially increase this tendency for de novo point mutations, particularly for mutations occurring in genes implicated in the RAS pathway [39,40]. Indeed, such mutations stimulate selfish selection during spermatogenesis in a process close to oncogenesis, leading to an over-representation of spermatozoids carrying these alterations over time. The *NF1* gene seems to obey this rule, too [41,42]. In the present cases, segregation analysis of family 5 showed that patient II-6 inherited her mutated allele from her father, as she transmitted her maternal chromosome to her children, who were not affected. In family 1, haplotype analysis indicated that the de novo c.7084_7085insGA pathogenic variant occurred in the *NF1* paternal allele of patient IV-1. His father (III-6) was 35 years old at the time of conception.

## 5. Conclusions

These clinical reports highlight the primordial importance of a systematic approach to the clinical and molecular diagnoses of NF1 patients. Putative co-occurrence of two or more distinct *NF1* mutations in a pedigree should not be underestimated.

## Figures and Tables

**Table 1:** Clinical features of the patients with molecular diagnosis of NF1 in the six families.

Family	Patient	Sex	Age	CALS *	Freckling	Subcutaneous/Cutaneous NFs	Plexiform NFs	Lisch Nodules	OPG	Skeletal Abnormalities	Other	*NF1* Variant
1	IV-1	M	21	>6	No	Yes	No	Yes, bilateral	No	Mild scoliosis, *cubitus valgus*	Anemic nevus, learning disabilities, headaches, myopia	c.7084_7085insGA
	IV-3 ^#^	F	18	>6	Yes, Ax & In bilateral	Yes	No	Yes, bilateral	No	Mild scoliosis	Facial nevus, left hemiparesis, hyperopia, mild intellectual disability	c.1885G>A
2	II-1	M	41	>6	No	No	No	No	ND	None		c.6642-5del
	III-2	F	14	>6	No	No	No	No	ND	None		c.6642-5del
	III-3 ^#^	M	2	>6	Yes, Ax bilateral	Yes	Yes	Yes, bilateral	Yes	Scoliosis	Spinal NFs, xanthogranulomas, headaches, attention deficit disorder	c.4537C>T
3	III-1	F	20	>6	Yes, SM bilateral	Yes	Yes	No	No	Scoliosis		c.5749+5G>A
	III-2 ^#^	F	15	>6	Yes, Ax bilateral	No	No	Yes	Yes	None		c.2044C>T
4	III-2	F	26	>6	Yes, Ax bilateral	Yes	No	Yes, left	No	None	Short stature, low-set ears	c.5154dup
	III-5 ^#^	M	39	>6	Yes, Ax bilateral	Yes	No	Yes, bilateral	No	None	Brugada syndrome (paternal origin)	c.3314+2T>C
5	II-6 ^#^	F	23	>6	Yes, Ax	Yes	Yes	Yes, bilateral	No	Scoliosis, short stature	Breast cancer, headaches	c.3916C>T
	IV-1	M	18	>6	Yes	Yes	Yes	No	Yes	Pseudarthrosis, sphenoid wing dysplasia	Learning disabilities	c.2376del
6	I-2	F	45	>6	No	Yes	No	ND	ND	Scoliosis		Complete deletion
	II-1	M	14	>6	Yes	No	No	No	No	None		
	II-2 ^#^	F	10	>6	Yes	Yes	No	ND	ND	None	Hypertelorism, congenital coronarocardiac fistula	Complete deletion

* CALS over 5 mm in greatest diameter in prepubertal individuals and over 15 mm in greatest diameter in postpubertal individuals. ^#^ Propositus. CALS: café-au-lait spots; NFs: neurofibromas; OPG: optic pathway glioma; Ax: axillary; In: inguinal; SM: sub-mammal; ND: not documented.

**Table 2:** Variants features and classification according to the ACMG-AMP guidelines.

Gene	Variant	Exon	Protein	Consequence	Studied Sample	Techniques	ACMG-AMP classification	Evidence of Pathogenicity	Reference
*NF1*	c.7084_7085insGA	48	p.Asn2362Argfs*14	Frameshift	DNA + RNA	RT-PCR + Sanger	Pathogenic	PVS1+ PS2+PM2+PP4+PP5	Sabbagh et al., 2013 [6]
	c.1885G>A	17	p.Gln616Glyfs*4	Creation of a new 3′ splice site	DNA + RNA	RT-PCR + Sanger	Pathogenic	PVS1+PS2+PP3+PP4+PP5	Ars et al., 2003 [19]
	c.6642-5del	45	p.Phe2215Hisfs*6	Exon 45 skip	DNA + RNA	NGS + RT-PCR + Sanger	Pathogenic	PVS1+PM2+PP4	ND
	c.4537C>T	35	p.Arg1513*	Premature stop codon	DNA + RNA	NGS + RT-PCR + Sanger	Pathogenic	PVS1+PP4+PP5	Side et al., 1997 [20]
	c.5749+5G>A	39	p.Ser1850fs*2	Exon 39 skip	DNA + RNA	NGS + RT-PCR + Sanger	Pathogenic	PVS1+PS2+PM2+PP3+PP4+PP5	Pros et al., 2008 [21]
	c.2044C>T	18	p.(Gln682*)	Premature stop codon (predicted)	DNA	NGS + Sanger	Pathogenic	PVS1+PS2+PM2+PP4	ND
	c.5154dup	37	p.(Phe1719Ilefs*17)	Frameshift (predicted)	DNA	NGS + Sanger	Pathogenic	PVS1+PM2+PP4	ND
	c.3314+2T>C	25	p.?	Unknown	DNA	NGS + Sanger	Pathogenic	PVS1+PM2+PP4	ND
	c.3916C>T	29	p.Arg1306*	Premature stop codon	DNA + RNA	RT-PCR + Sanger	Pathogenic	PVS1+PS2+PP4+PP5	Park & Pivnick, 1998 [22]
	c.2376del	20	p.Asn793Thrfs*28	Frameshift	DNA + RNA	RT-PCR + Sanger	Pathogenic	PVS1+PS2+PM2+PP4+PP5	Sabbagh et al., 2013 [6]
*SPRED1*	c.944C>T	7	p.(Pro315Leu)	Missense (predicted)	DNA	NGS + Sanger	Uncertain significance	PP3	ND

PVS1: null variant (nonsense, frameshift, canonical ±1 or two splice sites) in a gene where loss of function is a known mechanism of disease; PS2: de novo variant with both maternity and paternity confirmed; PM2: absent in the general population (controls) in gnomAD and dbSNP; PP3: multiple lines of computational evidence support a deleterious effect on the gene or gene product (conservation, evolutionary, splicing impact); PP4: patient’s phenotype is highly specific for NF1; PP5: reputable sources (LOVD, ClinVar) report variant as pathogenic. ND: not described. An asterisk (*) refers to a stop codon. A question mark (?) refers to unknown protein consequences.

## References

[1] Williams V.C., Lucas J., Babcock M.A., Gutmann D.H., Korf B., Maria B.L. (2009). Neurofibromatosis type 1 revisited. Pediatrics.

[2] Ferner R.E., Huson S.M., Thomas N., Moss C., Willshaw H., Evans D.G., Upadhyaya M., Towers R., Gleeson M., Steiger C. (2007). Guidelines for the diagnosis and management of individuals with neurofibromatosis 1. J. Med. Genet..

[3] Tucker T., Wolkenstein P., Revuz J., Zeller J., Friedman J.M. (2005). Association between benign and malignant peripheral nerve sheath tumors in NF1. Neurology.

[4] Stumpf D.A. (1988). Neurofibromatosis. Conference statement. National Institutes of Health Consensus Development Conference. Arch. Neurol..

[5] Friedman J.M. (1999). Epidemiology of neurofibromatosis type 1. Am. J. Med. Genet..

[6] Sabbagh A., Pasmant E., Imbard A., Luscan A., Soares M., Blanché H., Laurendeau I., Ferkal S., Vidaud M., Pinson S. (2013). NF1 molecular characterization and neurofibromatosis type I genotype-phenotype correlation: The French experience. Hum. Mutat..

[7] Von Deimling A., Krone W., Menon A.G. (1995). Neurofibromatosis type 1: Pathology, clinical features and molecular genetics. Brain Pathol..

[8] Xu G., O’Connell P., Viskochil D., Cawthon R., Robertson M., Culver M., Dunn D., Stevens J., Gesteland R., White R. (1990). The neurofibromatosis type 1 gene encodes a protein related to GAP. Cell.

[9] Clementi M., Barbujani G., Turolla L., Tenconi R. (1990). Neurofibromatosis-1: A maximum likelihood estimation of mutation rate. Hum. Genet..

[10] Kluwe L., Siebert R., Gesk S., Friedrich R.E., Tinschert S., Kehrer-Sawatzki H., Mautner V.F. (2004). Screening 500 unselected neurofibromatosis 1 patients for deletions of the *NF1* gene. Hum. Mutat..

[11] Mautner V.F., Kluwe L., Friedrich R.E., Roehl A.C., Bammert S., Högel J., Spöri H., Cooper D.N., Kehrer-Sawatzki H. (2010). Clinical characterisation of 29 neurofibromatosis type-1 patients with molecularly ascertained 1.4 Mb type-1 NF1 deletions. J. Med. Genet..

[12] Pasmant E., Sabbagh A., Spurlock G., Laurendeau I., Grillo E., Hamel M.J., Martin L., Barbarot S., Leheup B., Rodriguez D. (2010). NF1 microdeletions in neurofibromatosis type 1: From genotype to phenotype. Hum. Mutat..

[13] Brems H., Chmara M., Sahbatou M., Denayer E., Taniguchi K., Kato R., Somers R., Messiaen L., De Schepper S., Fryns J.P. (2007). Germline loss-of-function mutations in SPRED1 cause a neurofibromatosis 1-like phenotype. Nat. Genet..

[14] Pasmant E., Sabbagh A., Hanna N., Masliah-Planchon J., Jolly E., Goussard P., Ballerini P., Cartault F., Barbarot S., Landman-Parker J. (2009). SPRED1 germline mutations caused a neurofibromatosis type 1 overlapping phenotype. J. Med. Genet..

[15] Brems H., Pasmant E., Van Minkelen R., Wimmer K., Upadhyaya M., Legius E., Messiaen L. (2012). Review and update of SPRED1 mutations causing Legius syndrome. Hum. Mutat..

[16] Wakioka T., Sasaki A., Kato R., Shouda T., Matsumoto A., Miyoshi K., Tsuneoka M., Komiya S., Baron R., Yoshimura A. (2001). Spred is a Sprouty-related suppressor of Ras signalling. Nature.

[17] Pasmant E., Parfait B., Luscan A., Goussard P., Briand-Suleau A., Laurendeau I., Fouveaut C., Leroy C., Montadert A., Wolkenstein P. (2015). Neurofibromatosis type 1 molecular diagnosis: What can NGS do for you when you have a large gene with loss of function mutations?. Eur. J. Hum. Genet..

[18] Richards S., Aziz N., Bale S., Bick D., Das S., Gastier-Foster J., Grody W.W., Hegde M., Lyon E., Spector E. (2015). Standards and guidelines for the interpretation of sequence variants: A joint consensus recommendation of the American College of Medical Genetics and Genomics and the Association for Molecular Pathology. Genet. Med..

[19] Ars E., Kruyer H., Morell M., Pros E., Serra E., Ravella A., Estivill X., Lázaro C. (2003). Recurrent mutations in the *NF1* gene are common among neurofibromatosis type 1 patients. J. Med. Genet..

[20] Side L., Taylor B., Cayouette M., Conner E., Thompson P., Luce M., Shannon K. (1997). Homozygous inactivation of the *NF1* gene in bone marrow cells from children with neurofibromatosis type 1 and malignant myeloid disorders. N. Engl. J. Med..

[21] Pros E., Gómez C., Martín T., Fábregas P., Serra E., Lázaro C. (2008). Nature and mRNA effect of 282 different NF1 point mutations: Focus on splicing alterations. Hum. Mutat..

[22] Park V.M., Pivnick E.K. (1998). Neurofibromatosis type 1 (NF1): A protein truncation assay yielding identification of mutations in 73% of patients. J. Med. Genet..

[23] Klose A., Peters H., Hoffmeyer S., Buske A., Lüder A., Hess D., Lehmann R., Nürnberg P., Tinschert S. (1999). Two independent mutations in a family with neurofibromatosis type 1 (NF1). Am. J. Med. Genet..

[24] Upadhyaya M., Majounie E., Thompson P., Han S., Consoli C., Krawczak M., Cordeiro I., Cooper D.N. (2003). Three different pathological lesions in the *NF1* gene originating de novo in a family with neurofibromatosis type 1. Hum. Genet..

[25] Messiaen L., Yao S., Brems H., Callens T., Sathienkijkanchai A., Denayer E., Spencer E., Arn P., Babovic-Vuksanovic D., Bay C. (2009). Clinical and mutational spectrum of neurofibromatosis type 1-like syndrome. JAMA.

[26] Baralle D., Mattocks C., Kalidas K., Elmslie F., Whittaker J., Lees M., Ragge N., Patton M.A., Winter R.M., ffrench-Constant C. (2003). Different mutations in the *NF1* gene are associated with Neurofibromatosis-Noonan syndrome (NFNS). Am. J. Med. Genet. Part A.

[27] De Luca A., Bottillo I., Sarkozy A., Carta C., Neri C., Bellacchio E., Schirinzi A., Conti E., Zampino G., Battaglia A. (2005). *NF1* gene mutations represent the major molecular event underlying neurofibromatosis-Noonan syndrome. Am. J. Hum. Genet..

[28] Stevenson D.A., Viskochil D.H., Rope A.F., Carey J.C. (2006). Clinical and molecular aspects of an informative family with neurofibromatosis type 1 and Noonan phenotype. Clin. Genet..

[29] Hüffmeier U., Zenker M., Hoyer J., Fahsold R., Rauch A. (2006). A variable combination of features of Noonan syndrome and neurofibromatosis type I are caused by mutations in the *NF1* gene. Am. J. Med. Genet. Part A.

[30] Nyström A.M., Ekvall S., Allanson J., Edeby C., Elinder M., Holmström G., Bondeson M.L., Annerén G. (2009). Noonan syndrome and neurofibromatosis type I in a family with a novel mutation in NF1. Clin. Genet..

[31] Ekvall S., Sjörs K., Jonzon A., Vihinen M., Annerén G., Bondeson M.L. (2014). Novel association of neurofibromatosis type 1-causing mutations in families with neurofibromatosis-Noonan syndrome. Am. J. Med. Genet. Part A.

[32] Bahuau M., Houdayer C., Assouline B., Blanchet-Bardon C., Le Merrer M., Lyonnet S., Giraud S., Récan D., Lakhdar H., Vidaud M. (1998). Novel recurrent nonsense mutation causing neurofibromatosis type 1 (NF1) in a family segregating both NF1 and Noonan syndrome. Am. J. Med. Genet..

[33] Pasmant E., Amiel J., Rodriguez D., Vidaud M., Vidaud D., Parfait B. (2012). Two independent de novo mutations as a cause for neurofibromatosis type 1 and Noonan syndrome in a single family. Am. J. Med. Genet. Part A.

[34] Bertola D.R., Pereira A.C., Passetti F., de Oliveira P.S.L., Messiaen L., Gelb B.D., Kim C.A., Krieger J.E. (2005). Neurofibromatosis-Noonan syndrome: Molecular evidence of the concurrence of both disorders in a patient. Am. J. Med. Genet. Part A.

[35] Thiel C., Wilken M., Zenker M., Sticht H., Fahsold R., Gusek-Schneider G.-C., Rauch A. (2009). Independent NF1 and PTPN11 mutations in a family with neurofibromatosis-Noonan syndrome. Am. J. Med. Genet. Part A.

[36] Prada C.E., Zarate Y.A., Hagenbuch S., Lovell A., Schorry E.K., Hopkin R.J. (2011). Lethal presentation of neurofibromatosis and Noonan syndrome. Am. J. Med. Genet. Part A.

[37] Goriely A. (2016). Decoding germline de novo point mutations. Nat. Genet..

[38] Lázaro C., Gaona A., Ainsworth P., Tenconi R., Vidaud D., Kruyer H., Ars E., Volpini V., Estivill X. (1996). Sex differences in mutational rate and mutational mechanism in the *NF1* gene in neurofibromatosis type 1 patients. Hum. Genet..

[39] Goriely A., Wilkie A.O.M. (2012). Paternal age effect mutations and selfish spermatogonial selection: Causes and consequences for human disease. Am. J. Hum. Genet..

[40] Maher G.J., Ralph H.K., Ding Z., Koelling N., Mlcochova H., Giannoulatou E., Dhami P., Paul D.S., Stricker S.H., Beck S. (2018). Selfish mutations dysregulating RAS-MAPK signaling are pervasive in aged human testes. Genome Res..

[41] Snajderova M., Riccardi V.M., Petrak B., Zemkova D., Zapletalova J., Mardesic T., Petrakova A., Lanska V., Marikova T., Bendova S. (2012). The importance of advanced parental age in the origin of neurofibromatosis type 1. Am. J. Med. Genet. Part A.

[42] Dubov T., Toledano-Alhadef H., Bokstein F., Constantini S., Ben-Shachar S. (2016). The effect of parental age on the presence of de novo mutations—Lessons from neurofibromatosis type I. Mol. Genet. Genomic Med..

